# Plasma and adipose tissue level of angiopoietin-like 7 (ANGPTL7) are increased in obesity and reduced after physical exercise

**DOI:** 10.1371/journal.pone.0173024

**Published:** 2017-03-06

**Authors:** Mohamed Abu-Farha, Preethi Cherian, Irina Al-Khairi, Dhanya Madhu, Ali Tiss, Samia Warsam, Asma Alhubail, Devarajan Sriraman, Faisal Al-Refaei, Jehad Abubaker

**Affiliations:** 1 Biochemistry and Molecular Biology Unit Dasman Diabetes Institute, Kuwait City, Kuwait; 2 Clinical Services Department; Dasman Diabetes Institute, Kuwait City, Kuwait; 3 Tissue Banking Unit Dasman Diabetes Institute, Kuwait City, Kuwait; East Tennessee State University, UNITED STATES

## Abstract

**Objective:**

ANGPTL7 is a member of the Angiopoietin-like (ANGPTL) protein family that is composed of eight proteins (1–8). Increasing evidence is associating ANGPTL proteins to obesity and insulin resistance. The biological role of ANGPTL7 is yet to be understood except for a recently proposed role in the pathophysiology of glaucoma. This study was designed to shed light on the function of ANGPTL7 in obesity and its modulation by physical exercise as well as its potential association with lipid profile.

**Methods:**

A total of 144 subjects were enrolled in this study and finished three months of physical exercise. The participants were classified based on their BMI, 82 subjects were non-obese and 62 obese. ANGPTL7 levels in plasma and adipose tissue were measured by ELISA, RT-PCR and immunohistochemistry.

**Results:**

In this study, we showed that ANGPTL7 level was increased in the plasma of obese subjects (1249.05± 130.39 pg/mL) as compared to non-obese (930.34 ± 87.27 pg/mL) (*p*-Value = 0.032). ANGPTL7 Gene and protein expression levels in adipose tissue also showed over two fold increase. Physical exercise reduced circulating level of ANGPTL7 in the obese subjects to 740.98± 127.18 pg/mL, (*p*-Value = 0.007). ANGPTL7 expression in adipose tissue was also reduced after exercise. Finally, ANGPTL7 circulating level showed significant association with TG level in the obese subjects (R^2^ = 0.183, *p*-Value = 0.03).

**Conclusion:**

In conclusion, our data shows for the first time that obesity increases the level of ANGPTL7 in both plasma and adipose tissue. Increased expression of ANGPTL7 might play a minor role in the regulation of TG level in obese subjects either directly or through interaction with other ANGPTL protein members. Physical exercise reduced the level of ANGPTL7 highlighting the potential for targeting this protein as a therapeutic target for regulating dyslipidemia.

## Introduction

Angiopoietin-like proteins (ANGPTL) are a family of proteins that have structural similarity to angiopoietin proteins [1, 2]. Seven proteins have been initially grouped into this family (ANGPTL1-7) until recently another protein called ANGPTL8 has been added to the list [2]. All proteins have an amino-terminal coiled-coil domain as well as a carboxyl-terminal fibrinogen-like domain except ANGPTL8 which lacks the later domain [1–4]. ANGPTL proteins are not known to bind tyrosine kinase receptors such as Tie 1 and Tie 2 distinguishing them from angiopoietin proteins [5]. ANGPTL proteins have been shown to play different physiological roles in metabolism, inflammation and cancer [6–12]. Increasing evidence is connecting these proteins to obesity and insulin resistance [8, 13–15]. ANGPTL2 for example has been shown to associate with adiposity and insulin resistance as well as the development of type 2 diabetes [9, 16]. ANGPTL3, 4 and 8 on the other hand, have been shown to play a major role in regulating lipid metabolism through their inhibition of lipoprotein lipase [4, 17]. Similarly ANGPTL6 has been shown to be higher in subjects with metabolic syndrome and to positively associate with HDL level [18, 19]. The levels of ANGPTL8 has been recently shown to be higher in obese and diabetic subjects and to positively associate with insulin resistance and fasting blood glucose in none-diabetic subjects [14]. Also called Lipasin, RIFL, or betatrophin, this protein attracted attention as it has been suggested to play a role in beta-cell proliferation; a claim that has been later disputed [3, 20–23]. However, its interaction with ANGPTL3 has been shown to regulate triglyceride level in mice [4].

ANGPTL7 is a poorly studied member of the ANGPTL protein family that has been initially discovered in the stromal layer of the cornea [24]. Its level has also been shown to be elevated in glaucoma [25] and its overexpression increases the collagen expression level while, its induction by glucocorticoids caused the up-regulation of important glaucoma-related proteins including fibronectin, myocilin and MMP1 [26]. Taken together, this data suggested that the ANGPTL7 coordinates the trabecular meshwork’s extracellular matrix and its response to steroids [27]. On the other hand, ANGPTL7 has been associated with various cancers potentially through its interaction with the WNT/-beta-catenin signaling pathway [2]. It has been shown to play a role in hepatic cancer cell metastasis [28]. Even though other ANGPTL proteins family members have been implicated in obesity, insulin resistance and diabetes, no studies have looked at the role of ANGPTL7 under these conditions. This study was designed to study the expression level of ANGPTL7 in circulation and in subcutaneous adipose tissue at the mRNA and protein levels and to study the effect of physical exercise on its expression level as well as its association with clinical markers of obesity.

## Research design and methods

### Study population and ethical statement

In the present study, a total of 144 subjects were recruited to undergo three months of physical exercise as described previously [29]. Based on their Body Mass Index (BMI), a total of 82 subjects were classified into non-obese (19.5 and ≤ BMI < 30 kg/m^2^) and 62 subjects were obese (30 ≤ BMI < 40 kg/m^2^). The formula used to calculate BMI is the standard BMI formula: body weight (in kilograms)/height (in meters squared). All subjects signed a written informed consent before their participation in the study which was approved by the Ethical Review Board of Dasman Diabetes Institute and abiding with the guideline ethical declaration of Helsinki. Subjects that were involved in any physical exercise within the last 6 months prior to this study were excluded as well as the morbidly obese subjects with BMI >40 kg/m^2^. Subjects with prior major illness or taking any medication and/or supplement known to influence the body composition or bone mass were also excluded from the study [29].

### Physical exercise program

Subjects underwent a supervised physical exercise program as described previously [29–32]. Briefly, the physical exercise program was supervised by the Fitness and Rehabilitation Center (FRC) of Dasman Diabetes Institute. Prior to physical exercise, participants were examined by CPET (symptom-limited maximal incremental cardiopulmonary exercise test) (COSMED Quark, Italy) using an electromagnetically braked cycle ergometer to determine the maximum heart rate (max HR) as well as the response to aerobic physical exercise as measured by the maximum oxygen consumption (V_O₂ Max_) for each subject. The physical exercise was a combination of both moderate intensity of aerobic and resistance training using either a treadmill or cycling. These sessions included a 10 minutes warm up and cooling down steps at 50–60% of max HR, along with 40 minutes of the prescribed physical exercise program at 65–80% of max HR. Subjects exercised three to five times per week at the recommended HR range. Strength training was performed two to three times a week according to the program plan. Physical exercise intensity, duration and blood pressures were recorded for each session. All trainings were supervised by qualified fitness professionals and a consultant respirologist from FRC as described previously [29–32].

### Blood collection and anthropometric and biochemical measurements

Blood samples were collected before starting the exercise and after the three months of the physical exercise period. Plasma was prepared using vacutainer EDTA tubes and then aliquoted and stored at -80°C until assayed as described previously [14, 29, 33]. Adipose tissue biopsies (about 1 g) were obtained from the periumbilical area by surgical biopsy after a local anesthesia as described previously [29]. Once removed, the biopsy was rinsed in cold PBS, divided into 4 pieces and stored at -80°C until assayed. The average of three blood pressure readings measured using an Omron HEM-907XL Digital sphygmomanometer were taken with a 5–10 minutes rest between each reading. Whole-body composition was determined by dual-energy radiographic absorptiometry device (Lunar DPX, Lunar radiation, Madison, WI). Fasting blood Glucose (FBG) triglyceride (TG), total cholesterol (TC), low density lipoprotein (LDL) and high density lipoprotein (HDL) were measured on the Siemens Dimension RXL chemistry analyzer (Diamond Diagnostics, Holliston, MA). Glycated hemoglobin (HbA1C) was determined using the Variant^TM^ device (BioRad, Hercules, CA).

### ANGPTL7 ELISA

The plasma samples were thawed on ice and centrifuged at 10000g for 5 minutes at 4°C to remove any debris as described previously [14, 29, 33]. Repeated freeze thaw cycles were avoided. ANGPTL7 levels in plasma were detected using the human ANGPTL7 ELISA Kit produced by Wuhan EIAAB Science Co. Ltd. ELISA kit was validated using recombinant ANGPTL7 spiked at known concentration into plasma. A plasma dilution of 1:4 showed linearity and was used in the assay. Intra-assay coefficients of variation were 5.6% to 8.6%, while the inter-assay coefficients of variation were 7.2% to 9.9%. No significant cross reactivity with other ANGPTL proteins was observed.

### Measurement of gene expression by Real-time quantitative PCR

Total RNA was extracted from frozen adipose tissue and PBMCs using RNeasy Lipid Tissue Mini Kit and AllPrep RNA/Protein Kit, respectively (Qiagen, Inc., Valencia, CA). Total RNA was isolated from PBMC and adipose tissue biopsies of obese non-diabetic (n = 8) and obese-diabetic (n = 8). The cDNA was prepared from total RNA sample using High Capacity cDNA Reverse Transcription Kits (Applied Biosystems, Foster City, CA). qRT-PCR was performed on Rotor-Disc 100 system using SYBR Green normalized to Gapdh (Qiagen, Inc., Valencia, CA). PCR primers used were: ANGPTL7 For., 5'-TAGAGATGGAGGACTG-GGAGG-3'; ANGPTL7 Rev., 5'- GTGCACACTTGTCCAAGCAG-3'; Gapdh For., 5′- AACTTTGGCATTGTGGAAGG-3’ and Gapdh Rev., 5′-TGTGAGGG-AGATGCTCAGTG-3’. Relative expression was assessed by using the ∆∆CT method [34].

### Immunohistochemistry and western blotting

Formalin fixed, paraffin embedded adipose tissue samples were prepared and used to make sections for immunohistochemical studies as described previously [29]. Briefly, subcutaneous superficial adipose tissue biopsies were surgically obtained from the periumbilical area after a local anaesthesia. After that, the biopsy was rinsed in cold PBS, paraffin embedded, sectioned and stored appropriately until assayed. Paraffin embedded adipose tissue sections were deparaffinized and the antigens were retrieved at high-temperature using antigen unmasking solution (Dako, Denmark). The endogenous peroxidase was quenched using 3% H2O2 (Merck Schuchardt, Gemany) for 60 min at RT. Sections were blocked with 5% fat-free milk for 60 min at RT followed by 1% BSA for another 60 min and then, incubated at 4°C for overnight with primary anti-ANGPTL7 antibodies (Abcam). After washing, sections were stained with horseradish conjugated secondary antibody (Dako, Denmark) for 60 minutes at RT. Colors were developed using DAB kit (Dako, Denmark) and sections were counterstained with hematoxylin (Sigma Aldrich, St. Louis, MO). Quantification of the immunohistochemical staining data was done using Aperio software version 6.3 (Molecular Devices, Downingtown, PA) with an established arbitrary threshold.

HepG2 and 3T3-L1 cell lines were obtained from American Type Culture Collection. Cells were maintained at 37°C in a 5% CO_2_ humidified incubator. HepG2 and 3T3-L1cells were treated overnight with 125 μM palmitate that was prepared in fatty acid free and low endotoxin Bovine Serum Albumin (Sigma Aldrich). Western blotting was performed as previously described [33]. Primary antibodies used were ANGPTL7he primary antibodies used in this study are raised against ANGPTL7 (Abcam), phospho-JNK and JNK (Cell Signalling Technology). GAPDH (Millipore) was used as a loading control. Protein bands were visualized by chemiluminescence and the images were captured using the Versadoc 5000 system (BioRad)

### Statistical analysis

Comparisons between non-obese and obese subjects before and after physical exercise were made by Student’s t-test. Spearman’s correlation coefficients were estimated to determine associations between ANGPTL7 and FBG measurements and biochemical variables. All data are reported as mean ± standard error of the mean (SEM). Statistical assessments were two-sided and considered to be significant when *p*-Value < 0.05. All analyses were performed using SAS (version 9.r; SAS Institute, Cary, NC).

## Results

### Study population characteristics at baseline and after exercise

Clinical and biochemical characteristics of the non-obese and obese subjects at baseline are shown in Table 1. Our population was made of 144 subjects including 82 non-obese and 62 obese subjects. The average age of non-obese participants was 40.44 ± 11.83 years while obese subject’s average age was 47.19 ± 12.69 years. Obese subjects had significantly higher BMI, percent body fat, soft lean mass and total body water, FBG, HbA1C, TG, TC, and LDL were also significant (*p*-Value < 0.05) while HDL was lower in obese subjects (Table 1). After physical exercise no significant difference has been observed for the non-obese subjects (Data not shown). Obese subjects on the other hand showed slight reduction in their BMI from 34.76 ± 3.22 to 33.36 ± 3.37 kg/m^2^ with a *p-V*alue of 0.099 as well as VO_2_ Max that showed significant improvement from 15.86 ±4.34 to 22.42 ± 4.48 (*p*-Value = 0.011) (Table 2).

**Table 1:** Characteristics of the non-obese and obese subjects included in this study.

Variable	Non-obese, Average ± SD, N = 82	Obese, Average ± SD, N = 62	p-Value
**Age (years)**	40.44 ± 11.83	47.19 ± 12.69	**0.001**
**BMI (kg/m**^**2**^**)**	24.66 ± 2.86	34.76 ± 3.22	**<0.001**
**Percent Body Fat**	31.58 ± 5.74	38.41 ± 5.76	**<0.001**
**Soft Lean Mass**	42.78 ± 8.75	54.26 ± 11.64	**<0.001**
**Total Body water**	33.59 ± 6.72	42.95 ± 8.93	**<0.001**
**VO**_**2**_**P**	21.89 ± 3.42	15.86 ± 4.34	**0.005**
**TC (mmol/L)**	4.98 ± 0.91	5.23 ± 0.94	0.112
**HDL (mmol/L)**	1.43 ± 0.48	1.21 ± 0.32	**0.001**
**LDL (mmol/L)**	3.08 ± 0.84	3.39 ± 0.89	**0.040**
**TG (mmol/L)**	0.93 ± 0.50	1.51 ± 1.00	**<0.001**
**FBG (mmol/L)**	5.11 ± 0.54	5.55 ± 1.04	**0.003**
**HbA1C (DCCT%)**	5.52 ± 0.41	5.83 ± 1.09	**0.040**

**Table 2:** Characteristics of the obese subjects before and after undertaking physical exercise.

Variable	Pre-Exercise Obese Average ± SD	Post-Exercise Obese Average ± SD	*p*-Value
**BMI (kg/m**^**2**^**)**	34.76 ± 3.22	33.36 ± 3.37	0.099
**Percent Body Fat**	38.41 ± 5.76	36.04 ± 5.84	0.127
**Soft Lean Mass**	54.26 ± 11.64	54.80 ± 9.52	0.842
**Total Body water**	42.95 ± 8.93	43.27 ± 7.32	0.878
**VO**_**2**_**P**	15.86 ± 4.34	22.42 ± 4.84	**0.011**
**TC (mmol/L)**	5.23 ± 0.94	5.11 ± 1.14	0.662
**HDL (mmol/L)**	1.21 ± 0.32	1.14 ± 0.25	0.334
**LDL (mmol/L)**	3.39 ± 0.89	3.35 ± 1.01	0.867
**TG (mmol/L)**	1.51 ± 1.00	1.44 ± 0.84	0.780
**FBG (mmol/L)**	5.55 ± 1.04	5.65 ± 1.06	0.715
**HBA1C (DCCT%)**	5.83 ± 1.09	5.85 ± 0.47	0.913

### Increased ANGPTL7 level in plasma and adipose tissue in obese subjects

**Fig 1:**
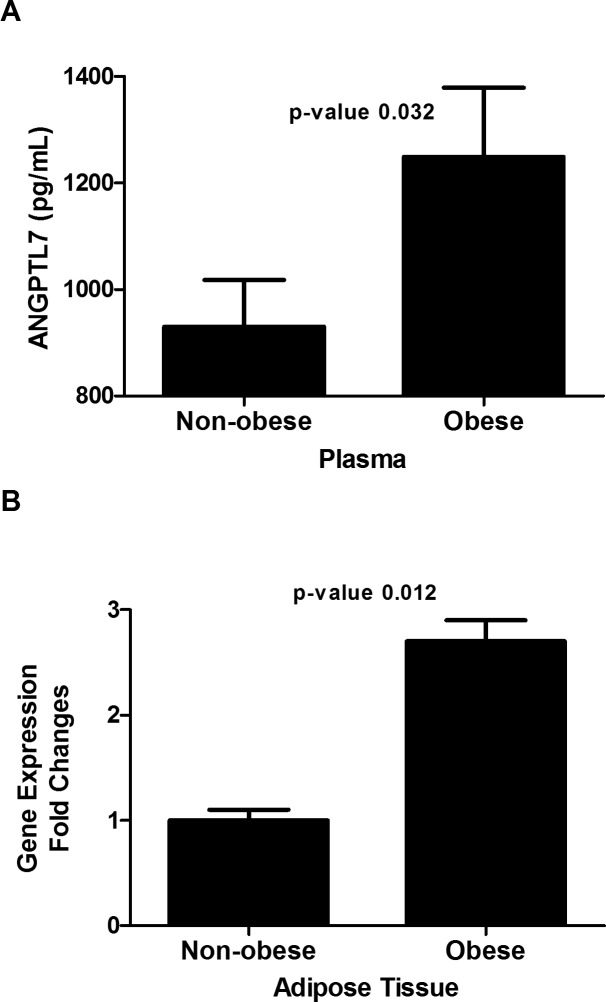
ANGPTL7 level in non-obese vs. obese. **A**: Plasma level of ANGPTL7 in non-obese (n = 82) vs. obese subjects (n = 62) adjusted for age, gender and ethnicity. **B**: Gene expression mRNA level of ANGPTL7 in adipose tissue isolated from non-obese (n = 8) vs. obese subjects (n = 8) expressed as fold changes. * *P-Value* < 0.05 as determined using student’s t-test.

Level of ANGPTL7 was examined in both plasma and adipose tissue of obese and non-obese subjects. Plasma level of ANGPTL7 adjusted for age, gender and ethnicity, showed higher levels in the obese subjects (1249.05± 130.39 pg/mL) vs. (930.34± 87.27 pg/mL) (*p*-Value = 0.032) for the non-obese subjects as shown in Fig 1A. Gene expression level in adipose tissue was measured using RT-PCR to study changes at the transcript level, mRNA levels of ANGPTL7 have shown a 2.7 fold increase in obese compared to the non-obese subjects (*p*-Value = 0.012) (Fig 1B).

### ANGPTL7 level in plasma and adipose tissue before and after physical exercise

**Fig 2:**
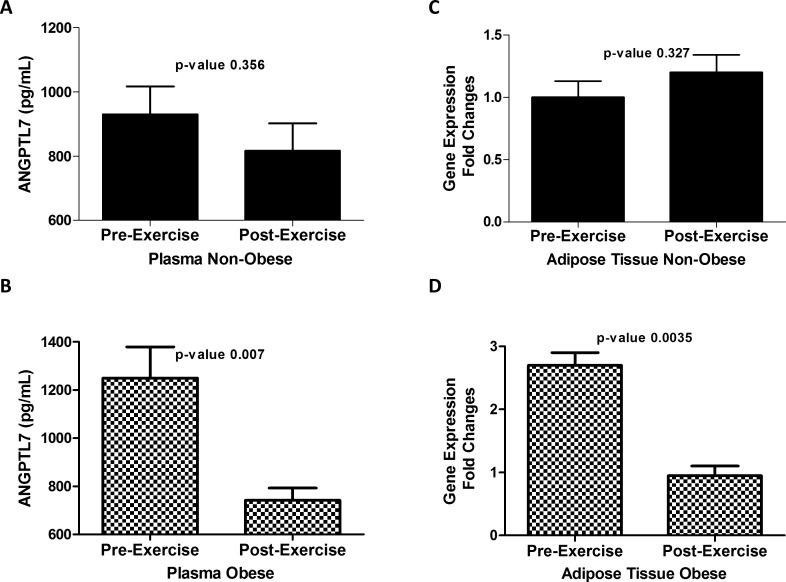
ANGPTL7 level after exercise. **A**: Plasma level of ANGPTL7 in non-obese subjects (n = 82) before and after three months of physical exercise adjusted for age, gender and ethnicity. **B**: Plasma level of ANGPTL7 in obese subjects (n = 62) before and after three months of physical exercise adjusted for age, gender and ethnicity. **C**: Gene expression level of ANGPTL7 in adipose tissue in non-obese subjects before and after three months of physical exercise expressed as fold changes. **D**: Gene expression level of ANGPTL7 in adipose tissue in obese (n = 8) subjects before and after three months of physical exercise expressed as fold changes. * *P-Value* < 0.05 as determined using student’s t-test.

Expression level of ANGPTL7 in plasma and adipose tissue of obese and non-obese subjects was examined before and after three months of physical exercise. Plasma level of ANGPTL7 adjusted for age, gender and ethnicity, was slightly reduced after physical exercise in the non-obese subjects without reaching statistical significance (930.34 ± 87.27 vs. 816.83±85.63 pg/ml, (*p*-Value = 0.356)) Fig 2A. However, plasma level of ANGPTL7 was significantly reduced in obese subjects after physical exercise (1249.05.04 ± 108.10 pg/ml) compared to (740.98 ± 127.18 pg/ml) after physical exercise (*p*-Value = 0.007) Fig 2B. A similar trend was also seen in the adipose tissue at the transcript level where ANGPTL7 level was reduced after physical exercise in the obese subjects (*p*-Value = 0.0035) but not in the non-obese subjects (*p*-Value = 0. 327) Fi 2 C & D.

### Immunohistochemistry staining of ANGPTL7 in adipose tissue

**Fig 3:**
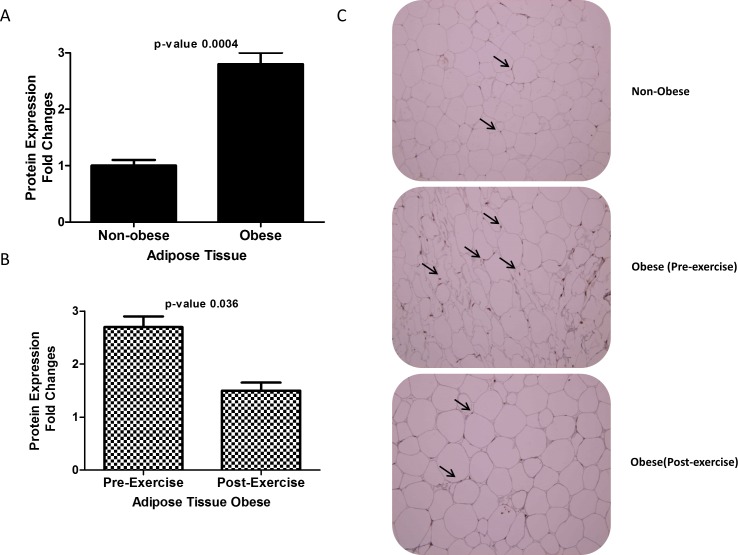
An Immunohistochemical staining for subcutaneous adipose biopsies from non-obese (n = 10) and obese (n = 10) non-diabetic participants using ANGPTL7 antibody. **B** Immunohistochemical staining of adipose tissue from obese subjects before exercise (n = 10) and after 3 months of exercise using ANGPTL7 antibody. Arrows indicate the positive staining. Aperio software was used for quantification and the values are illustrated at the bottom as fold changes after exercise. Student *t-test* for two group analysis was done to compare ANGPTL7 expression ^between^ non-obese, Paired t-test between obese before and after exercise. * *P*-Value < 0.05.

Paraffin embedded sections of adipose tissues from obese and non-obese subjects were stained to study the expression pattern of ANGPTL7. Similar to circulation levels, protein level of ANGPTL7 was increased over two folds in obese subjects compared to non-obese (*p*-Value = 0.0004) Fig 3A. Furthermore, following physical exercise a reduction in the level of ANGPTL7 in the obese subjects (*p*-Value = 0.036) was observed as shown in Fig 3B. Representative Immunohistochemistry sections from adipose tissues showing ANGPTL7 staining from non-obese and obese before and after exercise are shown in Fig 3C.

### Correlation between ANGPTL7 and TG

**Fig 4:**
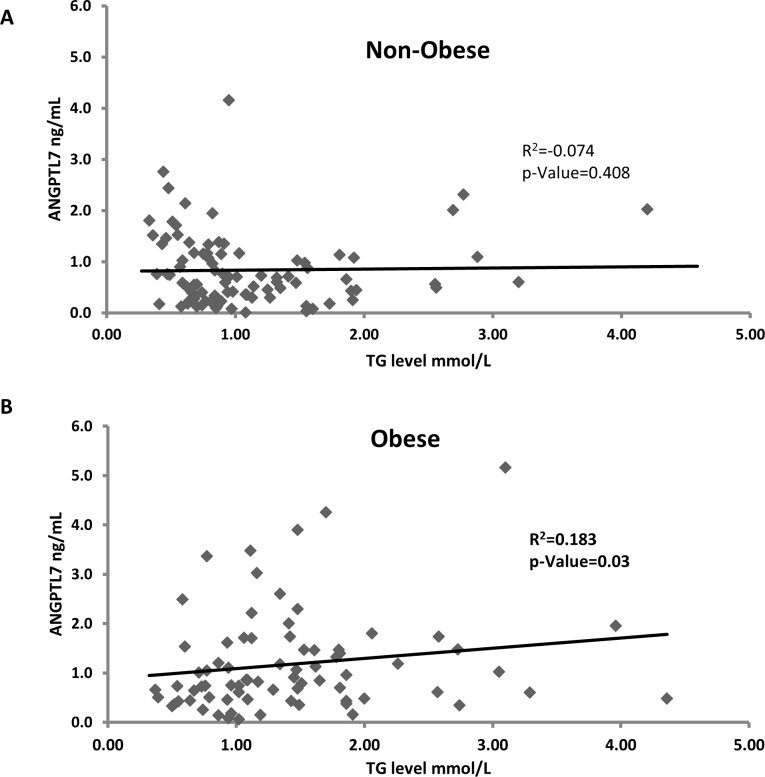
Spearman’s correlation between circulation level of ANGPTL7 and TG in the non-obese and the obese adjusted for age, gender and ethnicity.

To better understand the role of ANGPTL7 in obesity we looked at its association with lipid profile. No association was observed between TG and ANGPTL7 level in the non-obese subjects R^2^ = 0.074, *p*-Value = 0.408 (Fig 4A). However, ANGPTL7 showed significant association with TG level in the obese subjects R^2^ = 0.183, *p*-Value = 0.03 (Fig 4B).

### Increased ANGPTL7 after palmitate treatment

**Fig 5:**
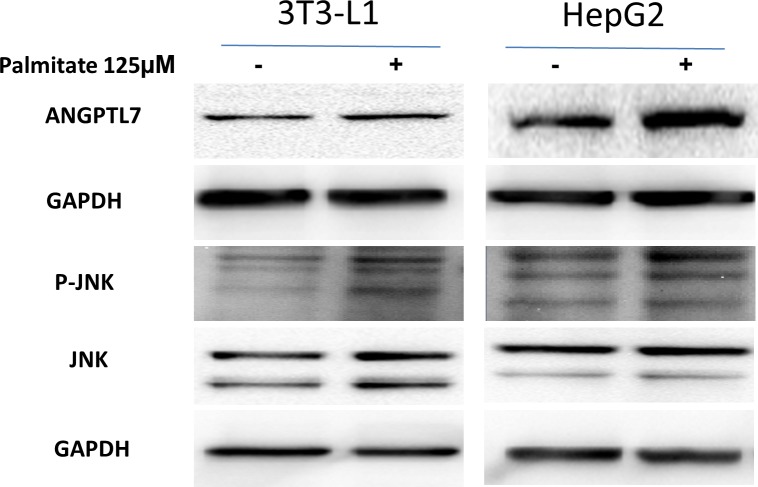
Immunoblotting data from HEPG2 and 3T3-L1 cells showing increased ANGPTL7 protein expression and phospho-JNK after 125 μM palmitate treatment overnight compared to untreated controls.

In order to mimic obesity conditions to test ANGPTL7 level in vitro, HepG2 and 3T3-L1 cells were treated with palmitate. As shown in Fig 5, ANGPTL7 level was increased in both cells after palmitate treatment. GAPDH was used as a loading control. JNK was also used as a control for the effectiveness of palmitate at inducing its phosphorylation. As shown in Fig 5, phosphor-JNK is induced after palmitate treatment in both cell lines.

## Discussion

In this study we show for the first time that the level of ANGPTL7 in circulation is increased in obese as compared to non-obese subjects. Similar data was also obtained in adipose tissues where mRNA and protein expression level were increased in the obese subjects. This increased ANGPTL7 level correlated with TG level in the obese subjects. A reduction in the level of ANGPTL7 in both plasma and adipose tissue in the obese subjects was seen following the exercise program but not in the non-obese subjects. Collectively, our data shed the light on the expression pattern of ANGPTL7 in obesity and its potential role in TG metabolism as well as the benefit of exercise in reducing its level in obese subjects. We also showed in vitro that ANGPTL7 level was increased after palmitate treatment.

ANGPTL7 is a member of the ANGPTL protein family that has been shown to play various roles in key pathways such as lipid metabolism, cancer and stem cell [2, 5, 9, 19]. They are secreted glycoproteins that are named after their sequence homology with angiopoietins [2]. All members of this family except for ANGPTL8 are structurally characterized by having an amino-terminal coild-coil domain connected by a linker region to a carboxy-terminal fibrinogen-like domain [2]. ANGPTL8 lacks the c-terminal fibrinogen-like domain [35]. ANGPTL7 is one of the least studied members of this family that has been mainly shown to be involved in myeloid cells metastasis and its level was down regulated in cancer cells [28]. Like other ANGPTL proteins, ANGPTL7 is located within the intron of another gene, intron 28 of FRAP1 gene encoding mammalian target of rapamycin (mTOR) protein [2]. In support of our data, a recent meta-analysis of 14 North American, Australian and European studies consisting of 5,530 cases and 8,318 controls from European ancestry showed that rs2300095 at MTOR-ANGPTL7 was associated with childhood obesity [36]. The authors highlighted the importance of this locus despite the fact that it was not replicated in the main defined overall pediatric setting as they may play a role in the pathogenesis of obesity as a whole [36].

ANGPTL7 has also been shown to be an important regulator of both human hematopoietic stem and progenitor cell expansion and regeneration [37, 38]. In this study we show that ANGPTL7 was increased in obese subjects and potentially linking it to TG plasma level. Other members of the ANGPTL protein family has been linked to obesity such as ANGPTL3, 4, 6 and 8 [2, 15]. We have recently showed that ANGPTL8 was increased in obesity and T2D and correlated with TG level in humans. It is possible that this increase in ANGPTL7 plasma level is connected to a potential role in TG level regulation as well as metabolism especially that its gene is located within the mTOR gene and the identification of rs2300095 at MTOR-ANGPTL7 linked with childhood obesity [36].

ANGPTL protein family members have emerged as an important regulator of TG level [12, 39–41]. The most prominent proteins involved in TG regulation are ANGPTL 3, 4 and the more recently discovered ANGPTL8 proteins [4, 9, 15, 42–46]. ANGPTL3 acts as a main regulator of lipoprotein metabolism through its interaction with lipoprotein lipase (LPL) [17, 47]. LPL enzyme is involved in the hydrolysis of TG from chylomicrons and VLDL lipoproteins [48]. ANGPTL3 activity is initiated by the proprotein convertase enzyme’s cleavage of its N-terminal domain that acts as an inhibitor of LPL activity [49]. Recent data showed that ANGPTL3 is most closely related to a recently identified member of this family called ANGPTL8 [4, 50]. ANGPTL8 has been shown to regulate ANGPTL3 role in lipid metabolism through interacting with its active N-Terminal domain. Mice lacking ANGPTL8 have been shown to have low TG level [4, 50]. The other key regulator of lipoprotein metabolism is ANGPTL4 which is considered a potent inhibitor of the LPL enzyme activity [2]. Even though, ANGPTL3, 4 and 8 might share common features in their regulation of lipoproteins, significant differences exist in their regulation of TG metabolism as well as their expression. ANGPTL3 and 8 regulate lipid metabolism in the fed state while ANGPTL4 inhibit its activity in the fast state [3, 51–54]. ANGPTL3 is exclusively expressed in the liver while ANGPTL4 and 8 are ubiquitously expressed [2]. In this study we show that ANGPTL7 is correlating weakly correlated with TG level in plasma, and might play some role in regulating its level directly or indirectly. ANGPTL7 level was also increased after palmitate treatment in vitro further supporting its role in obesity. A recent conference abstract was also published showing increased ANGPTL7 level in obesity [55]. Nonetheless, more data is required to further support this correlation and its functional role in this pathway.

Physical exercise has been recognized as a key positive modulator of obesity associated metabolic pathways such as insulin signalling and inflammation [56–61]. Exercise has been shown to increase the activity of proteins involved in metabolism such as AMPK [62–64] and reduces the expression of inflammation associated proteins such as TNFα [65–67]. For example, we have previously showed that exercise is an important regulator of DNAJB3 protein a member of the HSP40 protein family that has been shown to be downregulated by obesity and upregulated after physical exercise. Its overexpression has been associated with improvement in glucose uptake and insulin signalling [29, 33]. In this study, we show that ANGPTL7 is downregulated after physical exercise in plasma and circulation. This is an important observation that further supports the role of this protein in regulating TG level in plasma and could also suggest that it might be involved in inflammation or other processes that are negatively affected by exercise. Future studies to look at the association between ANGPTL7 and inflammatory markers will be important to better study its function as well as its association with other ANGPTL proteins to better understand its role in TG metabolism and other lipoproteins.

In conclusion, we have demonstrated for the first time that the expression of ANGPTL7 is increased in obesity in circulation as well as in adipose tissue. The increased ANGPTL7 expression is positively associated with TG level in obese subjects. Increased ANGPTL7 level in obese subjects was reduced after physical exercise. Taken together, ANGPTL7 might constitute another target for obesity therapeutic drugs that can be further studied to better understand its role in lipid metabolism and other metabolic pathways.
